# Energy Consumption Modeling of 3D-Printed Carbon-Fiber-Reinforced Polymer Parts

**DOI:** 10.3390/polym15051290

**Published:** 2023-03-03

**Authors:** Akash Shashikant Tiwari, Sheng Yang

**Affiliations:** School of Engineering, University of Guelph, Guelph, ON N1G 2W1, Canada

**Keywords:** energy consumption modeling, carbon-fiber-reinforced polymer, fused deposition modeling, design of experiments, sustainability

## Abstract

Three-dimensionally printed carbon-fiber-reinforced polymer (3DP-CFRP) has become an important contributor to commercialized additive manufacturing. Due to carbon fiber infills, the 3DP-CFRP parts can enjoy highly intricate geometry, enhanced part robustness, heat resistance, and mechanical properties. With the rapid growth of 3DP-CFRP parts in the aerospace, automobile, and consumer product sectors, evaluating and reducing their environmental impacts has become an urgent yet unexplored issue. To develop a quantitative measure of the environmental performance of 3DP-CFRP parts, this paper investigates the energy consumption behavior of a dual-nozzle fused deposition modeling (FDM) additive manufacturing process which includes melting and deposition of the CFRP filament. An energy consumption model for the melting stage is first defined using the heating model for non-crystalline polymers. Then, the energy consumption model for the deposition stage is established through the design of experiments approach and regression by investigating six influential parameters comprising the layer height, infill density, number of shells, travel speed of gantry, and speed of extruders 1 and 2. Finally, the energy consumption models are combined and experimentally tested with two different CFRP parts. The results show that the developed energy consumption model demonstrated over 94% accuracy in predicting the energy consumption behavior of 3DP-CFRP parts. The developed model could potentially be used to find a more sustainable CFRP design and process planning solution.

## 1. Introduction

Three-dimensionally printed carbon-fiber-reinforced polymer (3DP-CFRP) composites have been receiving rapidly increased attention due to their intricate geometry structures, attainable high mechanical strength, and superior electromagnetic properties [1]. In typical CFRP composites, the carbon fiber is used as an infill to support the load, while the matrix material (usually thermoplastic, such as acrylonitrile-butadiene-styrene (ABS)) helps to bind, protect, and transfer loads to the fibers. Many innovative applications of these CFRPs have been seen in aerospace applications, automobiles, wind energy applications, and surgical tools [2].

The fused deposition modeling (FDM) process remains one of the most popular additive manufacturing (AM) methods for fabricating CFRP because of its low cost and flexibility in modifying material compositions and mechanical properties. FDM is a process where a filament is melted using heat energy and deposited layer by layer as per the 3D model data. Through the FDM process, both short fibers [3] and continuous fibers [4] have been reported in various studies. Most work with 3DP-CFRP has focused on improving the mechanical properties or developing analytic models for predicting the mechanical properties [1], while rare research has been dedicated to understanding or improving the sustainability of 3DP-CFRP parts. Some pioneering work tried to recycle the carbon fiber content after the printing process [5], develop a more sustainable replacement for carbon fibers [6], or compare the life cycle impacts of AM and traditional molding for CFRP production [6]. In these studies, energy consumption in the fabrication process is typically characterized by using specific energy consumption (e.g., MJ/kg) and mass. This approach is appropriate for characterizing the life cycle performance of CFRP parts because of the large scope, but it does not have sufficient resolution to differentiate the amount of energy consumed between two parts that have the same mass but different geometry and process parameters. Such a high-resolution energy consumption model is yet established but critical for finding a more energy-efficient solution for 3DP-CFRP parts.

In order to develop a predictive model for the energy consumption behavior of 3DP-CFRP, an FDM process with dual nozzles that support continuous fiber is used as a research object. To achieve this goal, the energy modeling of both FDM processes and other AM processes are first reviewed. Then, a methodological framework for developing the predictive energy consumption model is proposed in Section 3. In this study, an experiment-based approach is adopted to derive the energy consumption model for the deposition stage of melted CFRP mixer, while a theoretical heating model is adopted for the melting stage of the CFRP. Section 4 introduces the experiment setup for the energy consumption model regarding printing process parameters. Finally, the energy consumption models for the melting stage and the deposition stage are combined and validated with two different 3DP-CFRP parts.

## 2. Literature Review

Research on the energy consumption of AM processes has been reported for various processes, including FDM, selective laser sintering (SLS), binder jetting, and stereolithography (SLA). These studies mostly focus on understanding the relationship between various process parameters and their impacts on electricity demands of the printers.

Process breakdown is popular for analyzing the energy consumption of various stages of FDM processes. Balogun et al. [7] broke the printing process into the start-up, warm-up, ready, and build stages. Electricity demand of each stage was measured, and it was found that energy consumption in the warm-up stage could account for over 73% for the first print cycle in FDM, but this value was significantly reduced in the following cycles, and the build stage dominated the power consumption. Similarly, Simon et al. [8] particularly investigated the impacts of printing speed and material flow rate on energy consumption of different FDM stages. Their results identified that the building stage was the most energy demanding one because of the long building time and that the material flow rate has a minor impact on power. 

The impacts of process parameters and utilization rate on energy consumption behavior have also been widely analyzed for FDM processes. Mognol et al. [9] investigated various building orientations and the positions of parts in the building chamber by experimentally measuring the electricity used in two different machine states, which include the idle and working conditions. Their results have shown that proper optimization of layer thickness or building orientation helps to reduce manufacturing time significantly, which consequently lowers energy consumption. Amongst the investigated FDM, SLS, and material jetting processes, FDM is the most energy efficient in both stand-by and in-work status for the same single printing job. To characterize the effects of single prints and multiple prints on energy consumption, Baumers et al. [10] designed a standard test part and measured the electricity in different machine capacities. It is interesting that full capacity utilization of FDM process does not significantly improve the energy savings compared to one-by-one printing. This is because minor changes were observed in the warm-up and cool-down stages. Peng and Yan [11] studied the effects of layer thickness, printing speed, and infill ratio on energy consumption and surface roughness for FDM printers. Analysis of variance of the results of the full factorial design showed that layer thickness is the most influential factor for energy consumption and secondary by infill ratio. Faludi et al. [12] showed that utilization profile imposes significant impacts on energy consumption behavior. Compared to low machine utilization (e.g., one job/week), maximized tool usage could potentially reduce ecological impacts by a factor of ten. Nguyen et al. [13] developed an energy estimation model by considering the power of the electric components of the FDM printer which includes step motor, heater, heat-bed, and auxiliary components (e.g., fans and controller). Their study showed that the most energy is consumed by the heating procedure. By introducing a new printing bed with controllable heating area, the energy savings could be as much as 23% compared to a traditional full-scale heating strategy.

Energy consumption research on other AM processes is also discussed. Sreenivasan and Bourell analyzed the energy consumption of various components of an SLS process. They found that the chamber heater consumes the most energy, followed by the roller drives, the laser transmitter, and the stepper motor. Later, they came up with a suggestion to improve the heat management system of a device or to use an energy-efficient transmitter [14]. To develop a comprehensive methodology for analyzing the energy consumption of various components in research work, Le Bourhis et al. [15,16] split the energy consumption into three parts: the cooling system, the motor drives, and the laser system. A predictive model was then developed to help assess environmental impact. Through Cooperative Effort on Process Emissions in Manufacturing (CO2PE!), Kellen et al. [17,18] described the parametric process model developed to estimate the ecological impact of SLS processes. Using various slice thicknesses and printing orientations, an empirical examination of the energy consumption of a stereolithography (SLA) method was conducted [19]. Meteyer and colleagues developed the binder-jetting process, including the calculation and validation of the energy and material usage. They tested the model with different widths, heights, and lengths of machines [20]. In another study, Xu and colleagues presented a model that considers the physical part’s relation to energy consumption [21].

From the above reviewed work on energy consumption of FDM and other AM processes, we can conclude that most of them tried to identify the key influential parameters for power demands, while rare efforts are focused on developing a predictive energy consumption model. Second, these studies typically consider the printing process (including warm-up, ready, building, and cooldown) as a whole to derive specific energy consumption (SEC) for the dedicated AM process. The SEC is measured by joule per kg, which lacks resolutions in the building stage that could potentially differentiate the energy performance of various design solutions. Lastly, no work is found to understand the energy behavior of 3DP-CFRP in FDM process yet.

## 3. Methodology

### 3.1. The Methodological Framework of Developing a Predictive Energy Consumption Model for 3DP-CFRP

**Figure 1:**
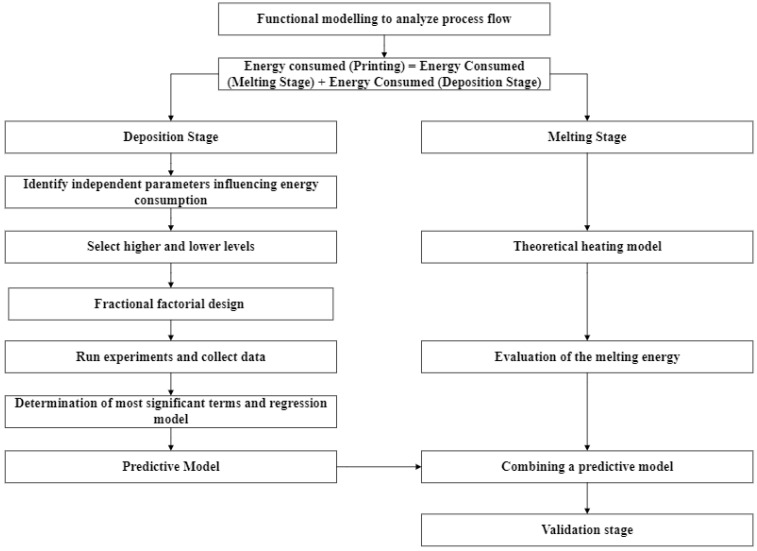
The proposed framework for developing a predictive energy consumption model of 3DP-CFRP.

The methodological framework of developing a predictive energy consumption model for 3DP-CFRP parts that are fabricated by FDM process is proposed in Figure 1. The framework starts with functional decomposition of the FDM process. This decomposition step helps to develop a better understanding of the subprocesses and analyze the critical stages of the complete workflow from machine startup to cooldown after the printing process. Studying all subprocesses is very time-consuming and challenging because commercial 3D printers are well self-contained, and the access to internal functional units such as motors, fans, controllers, etc., is very limited. As such, this study will only choose the actual printing phase (i.e., the melting of CFRP and decomposition of CFRP on the printing bed) as the focus for studying energy consumption behavior. Details of functional modeling and decomposition of the process are discussed in Section 3.2. 

The printing mechanism is examined using the process breakdown study, and it is inferred that the printing mechanism consists of two subprocesses. The first subprocess is the melting stage where filaments are molten into liquid, and the second stage is the deposition stage where the molten material gets deposited according to the geometry information. Hence, the energy consumption model consists of two parts: energy consumption models at melting and deposition. In the deposition stage, energy consumption behavior regarding a CFRP part is characterized by empirical values. Independent process parameters that influence energy consumption behavior are first identified. By determining the feasible ranges (i.e., high level and low level) of each process parameter, a design of experiments (DOE) approach/fractional factorial design is utilized to study the impacts of each parameter. Experiments are conducted to collect data of the energy consumption under various combinations of process parameters. Then, a regression model is developed as the predictive model for energy consumption in the deposition stage. In the melting stage, a theoretical heating model based on non-crystalline polymers is introduced. Afterwards, the energy consumption models in both the deposition and melting stages are combined as a joint predictive model. The accuracy of the joint predictive model is further validated with experiments of different parts.

### 3.2. Functional Modeling of the FDM Process

**Figure 2:**
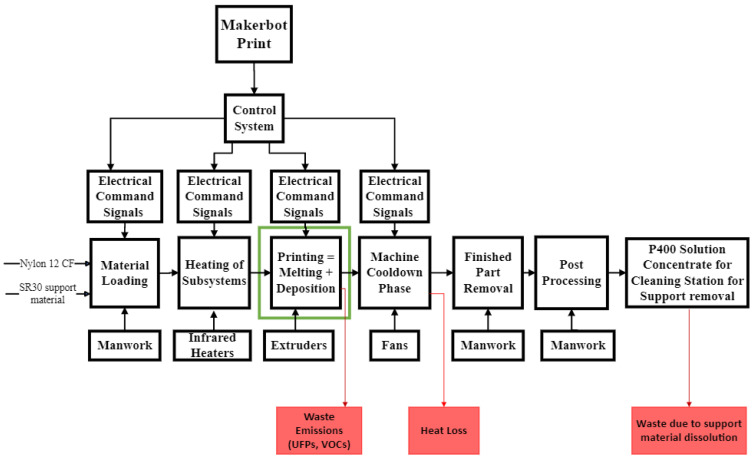
Functional decomposition of the fused deposition modeling process.

In the FDM process, energy is required to deliver the filament material to the various stages of the process. This includes establishing proper atmospheric and thermal background conditions, the positioning and calibration of extruders, and the heating subsystems. The functional model of the FDM process is built using the IDEF0 (Integration Definition for Process Modeling) diagram as shown in Figure 2. The FDM process starts with the loading of materials with both part materials (Nylon 12 carbon-fiber composite) and support materials (SR30). Then, the heating of subsystems starts where the heater cartridge heats the extruder nozzles, build plate, and chamber to be ready for the actual printing stage. In the printing stage, filaments are first melted into liquid form and deposited on the printing bed with desired patterns. When the printing job is complete, cooling fans get activated and the heater is switched off. After the stipulated time, the 3D-printed part is safely removed from the chamber with the support material. The final stage is the support material removal from the fully formed part. The part is put into a chemical solution concentrate for support removal. Finally, the final part is ready for desired applications. It is noted that all the logic control is realized by the sub-control systems embedded in the CFRP 3D printer. Since the difference in energy consumption in the material loading, heating of subsystems, and machine cooldown phases is negligible for parts with different geometry or process parameters, this paper will particularly focus on the energy consumption model of the printing phase.

Taking the Makerbot^®^ MethodX CFRP printer as an example, the most influential parameters which affect the energy consumption during the printing stage include the layer height, infill density, number of shells, travel speed of the gantry, extruder 1 speed (solid), and extruder 2 speed (solid). The feasible ranges of each parameter are defined by two levels (i.e., low level and high level), as summarized in Table 1. These parameters will be further investigated with respect to corresponding energy consumption behavior. It should be noted that building orientation is not considered in this work because optimization of building orientation is a complex decision which may affect surface quality, mechanical strength, and printing time [22,23] beyond energy consumption considerations. In this paper, we assume that the building orientation is pre-determined by pursuing the best surface quality before the process planning stage. However, it will be interesting to expand the developed predictive model in the planned future work to find an energy efficient building orientation while securing the functionality and quality of 3DP-CFRP parts. 

### 3.3. Energy Consumption Modeling in the Deposition Stage

To build the energy consumption model in the deposition stage, a two-level fractional factorial design DOE approach is adopted considering the high and low levels for each parameter. Compared to the full factorial design, the fractional approach reduces the number of experiments from $2^{k}$ to only $2^{k-2}$, while it maintains the main effects of two-factor interactions [24]. Here, k represents the number of independent factors. Given the 6 independent factors identified for the power consumption of 3DP-CFRP in the previous section, a total number of 16 experiments are needed. Experimental data of the 16 trials are collected and analyzed using ANOVA (analysis of variance) to determine the most significant parameters. The power consumed vs. time graph was extracted from Power Log 430-II 5.2 software, and the area under the curve was used to obtain the power consumed during the deposition stage. The data collected during the experiment were analyzed using the Minitab software, Version 21.1.0. The various tools used in the analysis include the normal probability plots and the Pareto plots. They identify the larger significant terms, such as the two-factor interactions and the main effects. ANOVA is also used to estimate the statistical significance of the terms. The function was considered as a linear function due to the value of R-values that show its linearity, and a predictive model in terms of the linear equation was deduced using regression. The results were then used to analyze the data and determine the significant terms of the responses and power consumption. Plots of residuals were then analyzed to confirm the existence of the satisfying assumption.

### 3.4. Energy Consumption Modeling in the Melting Stage

The thermal energy required to break bonds in crystalline solids is not uniform due to the diversity of bond strengths. The strength of interatomic and intermolecular bonds can vary depending on various factors, such as the type of bond, the arrangement of atoms and molecules, and the electronic and structural characteristics of the material [25]. The exact amount of thermal energy required to break a bond depends on the bond strength and the properties of the material [25]. Nylon 12 carbon fiber is a non-crystalline material, so the thermal energy required to break these bonds is even more non-uniform due to abrupt arrangement of its atoms and molecules. The energy consumption during the melting stage was deduced through the literature review [26]. Hence, the melting energy is shown in Equation (1).

(1) $$EC_{Melting}=V\frac{Pv}{250\pi d^{2}}$$

where *V* is the volume of the printed material (mm^3^), v is the feed rate of the feed roller (mm/s), *D* is the diameter of the filament (mm), and *P* is the power of the heater cartridge (W).

## 4. Experiment Setup

**Figure 3:**
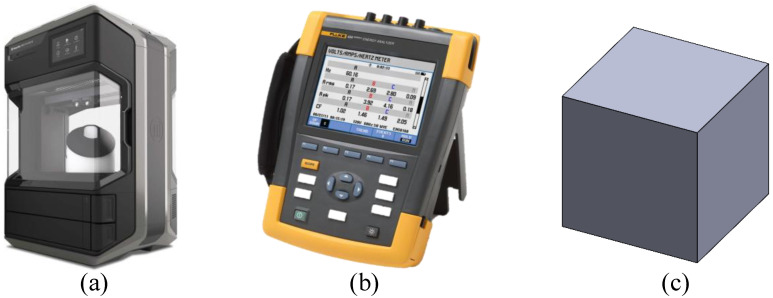
Experimental setup: (**a**) MakerBot method x carbon fiber edition [27], (**b**) Fluke 434 ii power analyzer [28], and (**c**) 3D model of a cube (10 mm × 10 mm × 10 mm).

MakerBot Method X Carbon-Fiber Edition (Model Number 900-0002A), an FDM-based CFRP printer, was used during the experiments, as shown in Figure 3a. It is one of the advanced 3D printers with its highly robust design, better reliability, and broader range of matrix material. The 3D printer consists of two extruders, a 1C extruder and a 2XA extruder, which print the matrix material and SR 30 support material, respectively. Nylon 12 carbon fiber was used as the model material for studying the energy consumption behavior of 3DP-CFRP. A Fluke 434 Series II power analyzer was used to monitor and record the energy consumption data in real time, as shown in Figure 3b. Power consumption was recorded in real time at intervals of 0.25 s. MakerBot print software was used for print preparation, including inputting the print parameters, slicing the model, estimating print time, and estimating material usage. A cube of 10 × 10 × 10 mm was used as the geometry for running the 16 trials of fractional factorial design of experiment. Although no support is needed for the cube, the 3D printer still adds one layer of support materials to increase adhesion of the part and the printing bed. The support type was chosen as Dissolvable–Tapered, which would significantly reduce PVA print and dissolution time. In the experiment, various process parameters from Table 1 were altered as per the runs. For example, in the first run (i.e., RUN 1) of the experiment, all the input parameters were run at a low level. Other parameters were kept constant for all the prints. Namely, the chamber temperature was set to 93 °C, extruder 1’s temperature was maintained at 280 °C, and extruder 2’s temperature was set to 180 °C.

**Figure 4:**
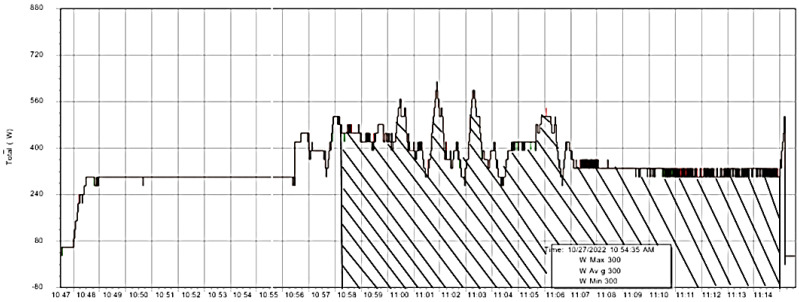
Experimental power vs. time graph in the RUN 1.

The real-time data for power consumption was recorded using the Fluke power analyzer and accessed using Power Log 430-II 5.2 software. The trend between power consumption regarding time was analyzed using the trapezoidal rule and the area under the curve was calculated. Figure 4 gives the real-time experimental power consumed (W) vs. time (s) for RUN 1. The approximate start and end times were determined using a stopwatch, which would be needed at power consumption during the printing phase. The area under the curve represents the actual energy consumption in the printing operation.

## 5. Results Analysis

A total of 16 experimental runs have been conducted according to the setting of the two-level fractional factorial design [24]. As shown in Table 2, A is the layer height (mm), B is the infill density (%), C is the number of shells, D is the travel speed of the gantry (mm/s), E is extruder 1 speed (mm/s), and F is the extruder 2 speed (mm/s). The factor selection process involves using a subset of basic factors known as A, B, C, and D. For the remaining factors, E and F, ABCE, and BCDF are the generators. The generators are the interactions of these factors, which give the remaining levels. The negative sign represents the lower level of the parameter in terms of the magnitude, while the positive sign represents the higher level as defined in Table 1. The energy consumption under each experimental condition is summarized in Table 2. For example, in RUN 1, with parameters of layer height (0.02 mm = lower level), infill density (20% = lower level), number of shells (2 = lower level), travel speed of gantry (100 mm/s = lower level), and extruder 1 speed (20 mm/s = lower level), the overall energy consumption during the printing stage is 250,088 Joules.

ANOVA analysis is conducted for the experimental data using Minitab software [29]. Table 3 shows the analysis of variance of the experimental values where the significance of each independent parameter is determined. The *p*-values for each independent parameter and its two-way interactions are calculated. The lower *p*-values imply the highest significance on the response variable. Layer height has the lowest *p*-value and a change in its value highly influences the response variable. On the other hand, the travel speed of the gantry has a higher *p*-value, which demonstrates that it has a relatively lesser influence on the output parameter. Similarly, regarding the two-way interactions, the layer height * infill density interaction implies that a considerable change in layer height and infill density will cause a significant change in the energy consumption even if the other independent parameters are unchanged.

**Figure 5:**
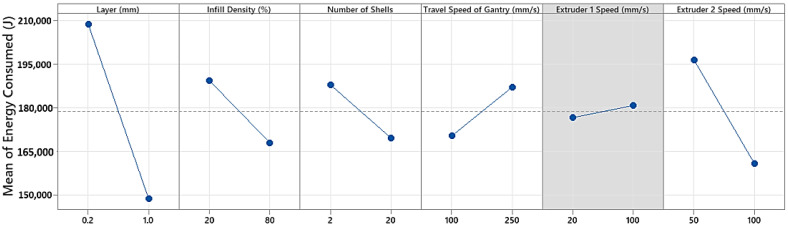
Main effects plot of energy consumption at the two levels.

The main effects plot is the graphical tool to determine the relative impact of various inputs on the energy consumption. The main effects plot (Figure 5) shows the energy outcome for each independent variable’s value. The above main effect plot clearly shows that the layer height of the material changes from 0.2 to 1.0 mm and the corresponding energy consumption decreases from 200,000 to 140,000 joules. Extruder 2’s speed, between 50 and 100 mm/s, shows a similar trend in energy consumption with a higher decreasing rate. Infill density and number of shells impose similar effects. With the increase of infill density and number of shells, the mean energy measured decreased to 167,000 joules. On the other side, as the gantry travel speed increases from 100 to 250 mm/s, the energy consumption increases from 170,000 to 185,000 joules. Extruder 1’s speed has fewer effects on energy consumption compared to other variables. Overall, layer height and extruder 2 speed contribute to a higher proportion in energy usage compared to the remaining parameters.

**Figure 6:**
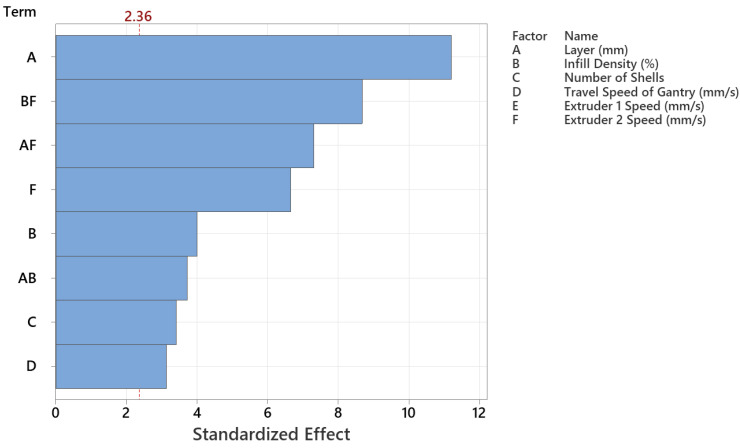
Pareto chart of the standardized effects.

**Figure 7:**
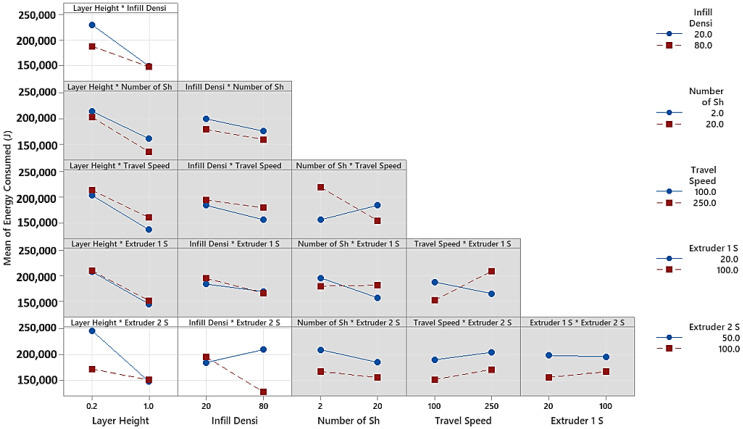
Interaction plot for two factors regarding energy consumption. Note: * indicates a combination of two parameters.

Pareto plots of the effects reveal the more prominent and likely significant effects. Significant terms deviate from the straight line near the normal effects plot’s center or exceed the Pareto chart’s threshold. The theory is that the insignificant terms are normally distributed based on the central limit theorem; hence, only terms deviating from that normal trend are likely significant. Independent parameter interactions (BF, AF, AB) are the terms that appear likely significant based on the effects shown in Figure 6. Interactions of extruder 2 speed and infill density or layer height are also effective compared to other variables. Based on the Pareto chart, layer height (A), the interaction of extruder 2 with infill density or layer height (BF and AF), extruder 2 speed (F), infill density (B), number of shells (C), and gantry travel speed (D) are the significant factors at a 5% significance level for the energy consumption. The interaction effects are shown in Figure 7, which implies that there exist significant interactions between layer height and infill density, layer height and extruder 2 speed, and infill density and extruder 2. However, all other interactions are insignificant regarding energy consumption.

**Figure 8:**
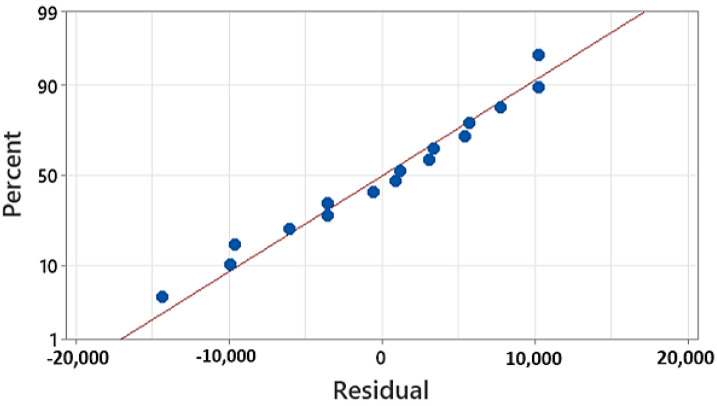
Residual plot for energy measured.

Figure 8 gives the residual plot for the measured energy demands, which provides information on the difference between the experimental value and mean value of all the 16 runs. Hence, according to the plotted points, the linear regression plot with the best fit is given in Figure 8. Therefore, the predictive energy consumption model is developed for the deposition phase as shown in Equation (2).

(2) $$EC_{Deposition}=284,092-264,896\times A+1475\times B-1023\times C+112.5\times D-341\times F+836\times A\times B+1969\times A\times F-31.15\times B\times F\;$$

where $EC_{Deposition}$ is the energy consumed during the deposition stage, *A* is the lay height, *B* is the infill density, *C* is the number of shells, *D* is the travel speed of gantry, and *F* is extruder 2 speed.

The joint energy consumption predictive model can be obtained by joining the models for melting (Equation (1)) and deposition (Equation (2)). The joint model is shown in Equation (3).

(3) $$EC_{Joint}=284,092-264,896\times A+1475\times B-1023\times C+112.5\times D-341\times F+836\times A\times B+1969\times A\times F-31.15\times B\times F+V\frac{Pv}{250\pi d^{2}}\;$$

Here, *A* = layer height (mm), *b* = infill density (%), *c* = number of shells, *d* = travel speed of gantry (mm/s), *f* = extruder 2 speed (mm/s), *V* is the volume of the printed material (mm^3^), v is the feed rate of the feed roller (mm/s), *D* is the diameter of the filament (mm), and *P* is the power of the heater cartridge (W).

## 6. Validation of the Joint Predictive Model

To validate the accuracy of the developed joint predictive model, two different examples—a coupling pin and a tripod design—are tested using the same Method X printer and Fluke for power measurement. Both designs were given the input as in the RUN-1 indicating all independent parameters at lower levels. More specifically, the printing parameters are layer height (0.02 mm = lower level), infill density (20% = lower level), number of shells (2 = lower level), travel speed of gantry (100 mm/s = lower level), and extruder 1 speed (20 mm/s = lower level). This provides a better analogy to compare the energy consumption while comparing these different geometries. The building orientation of both cases are predefined for the best surface quality (e.g., securing the surface finish for the holes in the tripod example). The chosen building orientations are indicated by the blue arrows in Table 4. For example, the building orientation of the tripod is along its central axis. 

The projected energy consumption is compared with the experimental measurement during the printing stage. As shown in Table 4, the projected energy consumption is very close to the experimental value in both examples with accuracy over 94%. This indicates that the developed predictive model is accurate to capture the energy consumption impacted by the six process parameters and changes in printed volume. As such, the joint predictive energy consumption model could be potentially used as a decision support tool for finding a more energy efficient design and process planning solution. 

## 7. Conclusions and Future Work

In conclusion, the present study provides a comprehensive investigation into the energy consumption behavior of 3DP-CFRP parts that are fabricated by the FDM process. The energy consumption model was found to consist of two key stages, deposition and melting, and the most critical parameters affecting the energy performance of the deposition stage were identified through a two-level fractional factorial design and ANOVA analysis. The main findings in this paper include the following.

(1) The results indicated that layer height, infill density, and speed of extruder 2 were the most important factors affecting the energy consumption behavior, while the speed of extruder 1 had the least effect.
(2) There exist significant interactions amongst respective parameter pairs, including layer height and infill density, layer height and extruder 2 speed, and infill density and extruder 2 speed. However, all other interactions are insignificant regarding energy consumption.
(3) A joint predictive model was established by combining the regression model and the melting energy, which was then validated through experimental results. The accuracy of the projected energy consumption was found to be over 94%.

This study represents a significant contribution to the field of energy consumption modeling for 3DP-CFRP parts and provides a better understanding of the complex relationship between design, process parameters, and energy consumption. The developed energy consumption model could be used to characterize the energy performance of various design solutions and process planning for 3DP-CFRP parts, thereby enabling the development of more energy efficient 3D printing processes. 

However, there are a few limitations to be acknowledged for the developed predictive model. First, the established predictive model is specific to nylon-based carbon fiber composites and the Makerbot MethodX printer. The generalization of methodological framework needs more investigation for other types of CFRP (e.g., epoxy-based composites) and FDM processes. Second, while two-factor interactions were tested in this study, future research into the impact of three-factor interactions may potentially further improve the prediction accuracy. Third, building orientation is considered as a pre-determined factor in this work, but it can be integrated as a variable for finding the energy-efficient building orientation while securing good printing quality in the planned next step. 

## Figures and Tables

**Table 1:** Independent parameters at two levels.

Factors	Description	Levels	
		Level I	Level II
Layer Height (mm)	Diameter of each deposited layer	0.02	0.1
Infill Density (%)	The density of the matrix material inside the outermost surface of the component	20	80
Number of Shells	Number of the outermost layer while printing	2	20
Travel Speed—Gantry (mm/s)	Speed of the gantry for X and Y axis movement	100	250
Extruder 1 Speed (mm/s)	Speed of 1C extruder that prints matrix material	20	100
Extruder 2 Speed (mm/s)	Speed of 2XA extruder that deposits SR 30 support material	50	100

**Table 2:** Recommended 2-level fractional factorial design with measured energy consumption.

Experiments	A	B	C	D	ABC = E	BCD = F	Energy Consumption (Joules)
RUN 1	−	−	−	−	−	−	250,088
RUN 2	+	−	−	−	+	−	128,250
RUN 3	−	+	−	−	+	+	128,700
RUN 4	+	+	−	−	−	+	117,277
RUN 5	−	−	+	−	+	+	205,508
RUN 6	+	−	+	−	−	+	153,150
RUN 7	−	+	+	−	−	−	230,790
RUN 8	+	+	+	−	+	−	148,097
RUN 9	−	−	−	+	−	+	218,700
RUN 10	+	−	−	+	+	+	202,215
RUN 11	−	+	−	+	+	−	260,610
RUN 12	+	+	−	+	−	−	197,179
RUN 13	−	−	+	+	+	−	244,283
RUN 14	+	−	+	+	−	−	113,400
RUN 15	−	+	+	+	−	+	131,963
RUN 16	+	+	+	+	+	+	128,453

**Table 3:** ANOVA of the experimental values.

Source	Degree of Freedoms	F Value	*p*-Value
Model	8	43.72	0.000
Linear	5	41.44	0.000
Layer Height (mm)	1	125.40	0.000
Infill Density (%)	1	16.02	0.005
Number of Shells	1	11.69	0.011
Travel Speed Gantry (mm/s)	1	9.80	0.017
Extruder 2 Speed Solid (mm/s)	1	44.26	0.000
2-Way Interactions	4	47.52	0.000
Layer Height (mm)*Infill Density (%)	1	13.87	0.007
Layer Height (mm)*Extruder 2 Speed Solid (mm/s)	1	53.44	0.000
Infill Density (%)*Extruder 2 Speed Solid (mm/s)	1	75.24	0.000

**Table 4:** Accuracy verification of the joint predictive model for two different parts.

Geometry (Orientation°)	Projected Power Consumption (Joules)	Experimental Power Consumption (Joules)	Volume of the Printed Material (mm^3^)	Accuracy (%)
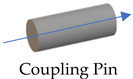	82,182.83	89,290.17	71,240.17	95.30
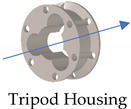	192,806.91	204,155.88	94,415.76	94.44

## Data Availability

Not applicable.
